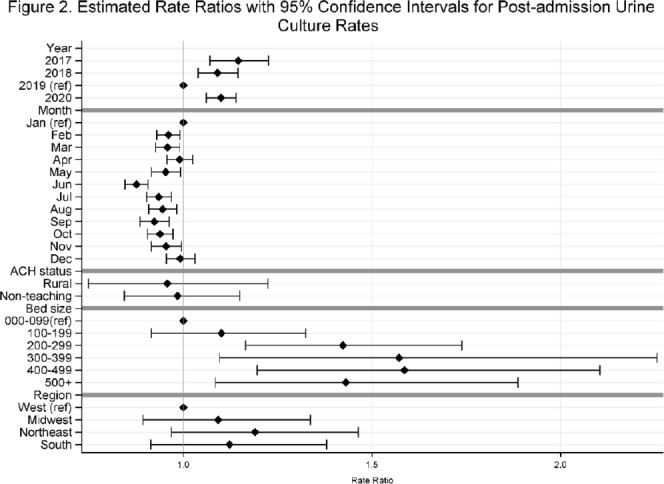# Temporal trends in urine-culture rates in the US acute-care hospitals, 2017–2020

**DOI:** 10.1017/ash.2022.75

**Published:** 2022-05-16

**Authors:** Sophia Kazakova, Natalie McCarthy, James Baggs, Kelly Hatfield, Babatunde Wolford, Babatunde Olubajo, John Jernigan, Sujan Reddy

## Abstract

**Background:** Previously, we reported decreasing postadmission urine-culture rates in hospitalized patients between 2012 and 2017, indicating a possible decrease in hospital-onset urinary tract infections or changes in diagnostic practices in acute-care hospitals (ACHs). In this study, we re-evaluated the trends using more recent data from 2017–2020 to assess whether new trends in hospital urine-culturing practices had emerged. **Method:** We conducted a longitudinal analysis of monthly urine-culture rates using microbiology data from 355 ACHs participating in the Premier Healthcare Database in 2017–2020. All cultures from the urinary tract collected on or before day 3 were defined as admission urine cultures and those collected on day 4 or later were defined as postadmission urine cultures. We included discharges from months where a hospital reported at least 1 urine culture with microbiology and antimicrobial susceptibility test results. Annual estimates of rates of admission culture and postadmission urine-culture rates were assessed using general estimating equation models with a negative binomial distribution accounting for hospital-level clustering and adjusting for hospital bed size, teaching status, urban–rural designation, discharge month, and census division. Estimated rate for each year (2018, 2019, and 2020) was compared to previous year’s estimated rate using rate ratios (RRs) and 95% confidence intervals (CIs) generated through the multivariable GEE models. **Results:** From 2017 to 2020, we included 8.7 million discharges and 1,943,540 urine cultures, of which 299,013 (15.4%) were postadmission urine cultures. In 2017–2020, unadjusted admission culture rates were 20.0, 19.6, 17.9, and 18.2 per 100 discharges respectively; similarly, unadjusted postadmission urine-culture rates were 8.6, 7.8, 7.0, and 7.5 per 1,000 patient days. In the multivariable analysis, adjusting for hospital characteristics, no significant changes in admission urine-culture rates were detected during 2017–2019; however, in 2020, admission urine-culture rates increased 6% compared to 2019 (RR, 1.06; 95% CI, 1.02–1.09) (Fig. 1). Postadmission urine-culture rates decreased 4% in 2018 compared to 2017 (RR, 0.96; 95% CI, 0.91–0.99) and 8% in 2019 compared to 2018 (RR, 0.92; 95% CI, 0.87–0.96). In 2020, postadmission urine-culture rates increased 10% compared to 2019 (RR, 1.10; 95% CI, 1.06–1.14) (Fig. 2). Factors significantly associated with postadmission urine-culture rates included discharge month and hospital bed size. For admission urine cultures, discharge month was the only significant factor. **Conclusions:** Between 2017–2019, postadmission urine-culture rates continued a decreasing trend, while admission culture rates remained unchanged. However, in 2020 both admission and postadmission urine culture rates increased significantly in comparison to 2019.

**Funding:** None

**Disclosures:** None

**Figure:**
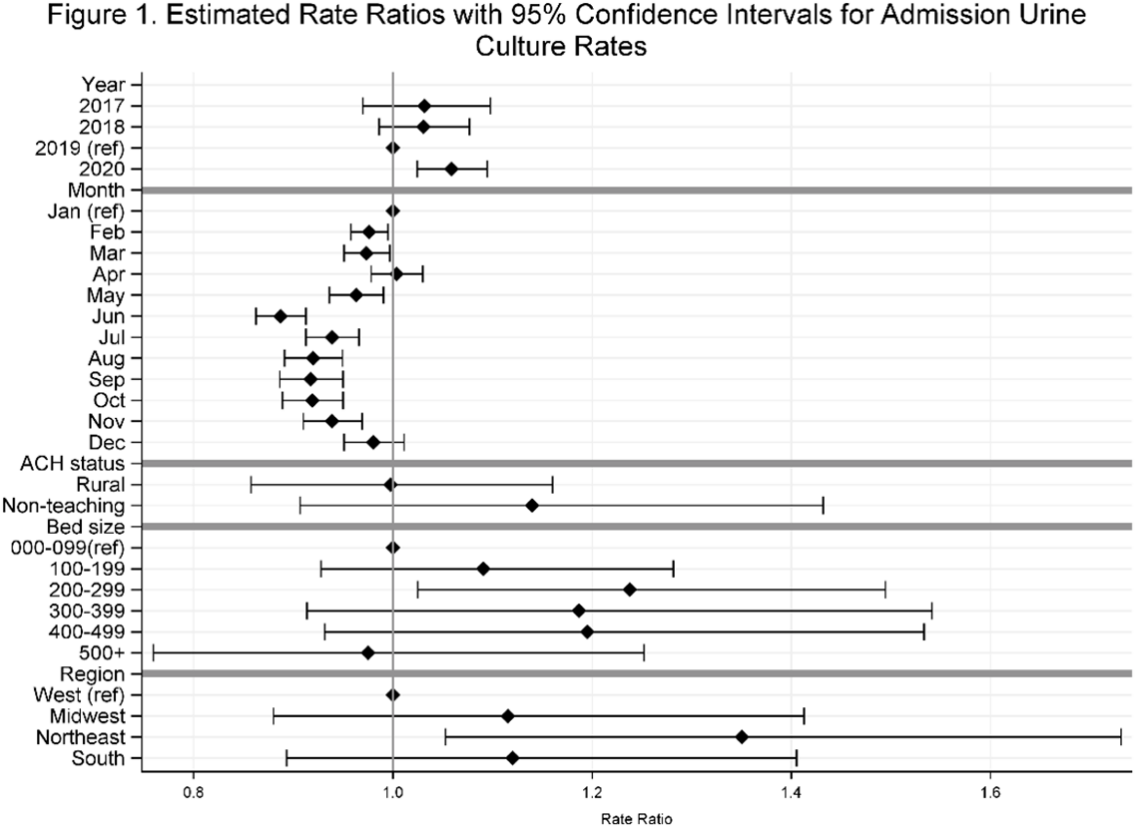

**Figure:**